# Sulfamethazine contamination level and exposure assessment in domestic and imported poultry meats in Jordan

**DOI:** 10.14202/vetworld.2019.1992-1997

**Published:** 2019-12-18

**Authors:** Saddam S. Awaisheh, Mohammad S. Khalifeh, Razan J. Rahahleh, Ja’far M. Al-Khaza’leh, Rania M. Algroom

**Affiliations:** 1 Department of Nutrition and Food Processing, Al-Balqa Applied University, Salt 19117, Jordan; 2 Department of Veterinary Basic Sciences, Jordan University of Science and Technology, Irbid 22110, Jordan; 3 Department of Food Science, Al-Balqa Applied University, Zarqa 19237, Jordan

**Keywords:** antimicrobial resistance, estimated daily intake, Jordan, poultry meat, sulfamethazine

## Abstract

**Background and Aim:**

Sulfamethazine (SMZ) is an important and widely used antibiotic in poultry industry due to its high efficacy in fighting diseases and promoting growth. In addition, SMZ is a possible human carcinogen and has been found in many food types including poultry meat. Accordingly, this study aimed to survey the contamination level and estimated daily intake (EDI) of SMZ in domestic and imported poultry meat samples in Jordan.

**Materials and Methods:**

A total of 120 samples; 60, 30, and 30 of fresh and frozen domestic and frozen imported poultry samples, respectively, were collected from different cities in Jordan. Poultry samples were analyzed for SMZ incidence rate and contamination level using a competitive enzyme-linked immunosorbent assay technique. EDI values were calculated from the SMZ concentration, average poultry daily consumption rate, and adult body weight (b.w.).

**Results:**

Of the 120 surveyed samples, 20 samples (16.7%) were SMZ violative positive and exceeded the European Union maximum limit (100 µg/kg) and accordingly were unfit for human consumption. Whereas, 51 samples (42.5%) were with SMZ concentrations of 10-100 µg/kg. The average SMZ concentration was 235.58 µg/kg, with a range of 11.47-800 µg/kg poultry meat. It is also noteworthy the high EDI of SMZ by Jordanian adults, 0.286 µg SMZ/kg b.w./day. Moreover, results prevailed that the highest SMZ incidence rate and contamination level were for imported poultry samples followed by domestic poultry samples, which may indicate that SMZ contamination in poultry meat is an international issue.

**Conclusion:**

The current study prevailed high SMZ incidence rate, contamination level, and EDI values, which is likely due to indiscriminate use of SMZ in poultry production. Results also prevailed the high risk that consumers in Jordan may expose due to SMZ residues. Therefore, more strict program and good agricultural practices should be applied to monitor antibiotic withdrawal periods in animals used for human consumption to ensure the legal residue requirements of these antibiotics.

## Introduction

Antibiotics are extensively used in poultry industry at subtherapeutic doses to improve meat production through increasing feed conversion, promoting growth rate, and preventing diseases [1,2]. Globally, it is estimated that 50% of the antibiotics produced in the world are used in animals as growth promoters [3]. Indiscriminate use of antibiotics often leads to the evolution of antimicrobial resistance (AMR) in pathogens, in both human and animal venues alike [4-6]. Prolonged exposure to subtherapeutic antibiotic doses leads to the proliferation of resistant bacterial strains, which might transfer AMR genes to other species of bacteria, with difficulty in predicting consequences to human health [7,8]. However, poultry industry is blamed for the dramatic rise and spread of AMR in bacteria [9-11]. However, AMR is a global health threat because it renders many antibiotics ineffective, and thus, simply treated infections may become more virulent and even deadly to humans soon [12]. In animal production, AMR can lead to more severe outbreaks of diseases and mass deaths among animal and poultry populations with enormous economic loses [6]. Besides the AMR crisis, antibiotic residues in animal foods may represent great health risks to humans due to its several side effects including toxicity, carcinogenicity, and sensitivity [13].

Sulfonamides (SAs) are a group of synthetic antibiotics with a broad-spectrum effect against the majority of G− and G+ bacteria. Due to its strong antimicrobial activity, SAs are used to treat several human infections such as respiratory and digestive tract infections. SAs have a bacteriostatic effect through binding ρ-aminobenzoic acid, which is necessary for folic acid synthesis and, consequently, inhibit bacterial DNA formation [14]. Among more than 5400 SA derivatives, sulfamethazine (SMZ) is one of the most used SAs in human and animal medication. SMZ is widely used by veterinarians in disease treatment and prevention or growth-promoting purposes in ruminants and poultry. The high efficacy and low cost have resulted in the wide use of SMZ in poultry production, as an additive in water or feed [15]. Several reports indicated that SMZ comprised approximately 95% of SA violations in animal tissues [9,16,17].

Chemical and microbiological safeties of poultry meat are of great concern for both consumers and legal authorities [18,19]. The European Union (EU) report concerning the chemical residues in animal foods showed that the SAs, including SMZ, are one of the most occurring and contaminating drugs [9]. SMZ is a suspected carcinogen [20] and has been detected and found in meat, fish, milk, and cheese [21-24]. Furthermore, SMZ is more heat stable than other SAs, which indicates that it is less affected by different cooking conditions and more residues left in cooked food [25]. Accordingly, the maximum residue limit (MRL) of SMZ in animal tissues is set at 100 ppb [26].

In Jordan, 60-70% of consumed meats are poultry meats [27]. Besides domestic production, Jordan imports poultry meat from different countries around the world. Few of these countries, like Europe, banned the use of antibiotics as growth promoters, while many other countries allow it. Moreover, the high consumption rate of poultry meat per capita in Jordan may imply a greater potential risk of higher antibiotics exposure by consumers.

On extensive search, no available data were found about the incidence of SMZ in poultry meat in Jordan. For this reason, this work aimed to assess the exposure risk of Jordanian population to SMZ through the determination of the levels of SMZ in fresh and frozen domestic and imported poultry meat marketed in Jordan.

## Materials and Methods

### Ethical approval

This study did not involve the use of live animals, and hence, ethical approval was not required.

### Poultry samples collection

A total of 120 of fresh and frozen domestic and frozen imported poultry samples (60, 30, and 30 samples, respectively) (Table-1) representing the most traded brands from different cities in Jordan were randomly collected during February and November 2017. Samples were collected in sterile bags and transferred in icebox to the laboratory. On reception, collected samples were stored at −20°C freezer until the analysis.

**Table-1:** Sources and numbers of poultry meat samples collected from Jordanian markets for investigation of sulfamethazine residues.

Poultry source	Number of samples
Domestic poultry	
Fresh	60
Frozen	30
Imported poultry	
Frozen	30
Total	120

### Poultry samples preparation

Fat-free meat pieces were taken from the collected samples and homogenized by a mixer to a fine paste. A 5 g portion of each homogenized sample was mixed vigorously with 20 ml of acetonitrile/water mixture (84/16) for 10 min and centrifuged at 3000 g/10 min/15°C. A 3 ml portion of the supernatant was then diluted with another 3 ml of distilled water followed by addition of 4.5 ml of ethyl acetate and mixing for 10 min. This mixture was centrifuged at 3000 g/10 min/15°C. The superior ethyl acetate layer was then transferred into another centrifugal vial and was evaporated to dryness. Dried residue was dissolved in 1.5 ml of buffer 1 (provided with the kit), and for further degreasing, 1.5 ml of n-hexane was added and mixed for 5 min, then centrifuged at 3000 g/10 min/15°C. The upper hexane layer was completely removed, and a 50 µL quantity of the aqueous phase was used for SMZ determination using a specific enzyme-linked immunosorbent assay (ELISA) kit.

### Analysis of SMZ by competitive ELISA

Determination of SMZ residues was carried out using Ridascreen sulfamethazine competitive ELISA kit (Art No. R3001) from R-Biopharm (Darmstadt, Germany), with a detection limit of 18 ppb. For the preparation of standard curve, SMZ standard solutions of 0, 30, 90, 270, and 810 ppb concentrations were provided with the kit. Before use, the kit materials were brought to room temperature (RT) (20-25°C). Sample analysis was carried out according to the manufacturer’s instructions. Briefly: SMZ microwells plate was pipetted with 50 µL of samples extract or each standard solution (for standard curve preparation) in duplicate. Then, 50 µL of diluted enzyme conjugate solution was added to each well followed by addition of 50 µL of diluted antibody solution to each well. The plate was then mixed gently by shaking and incubated for 2 h at RT. The liquid was then poured out of the wells and the microwells plate tapped upside down vigorously for 3 times against absorbent paper to ensure complete liquid removal from the wells. The wells were then washed with 250 µL distilled water and the liquid was poured out again for 2 times. A 50 µL of substrate and 50 µL of chromogen were added to each well, and then, the plate was mixed by gentle shaking and incubated for 30 min in the dark at RT. Finally, 100 µL of the stop solution was added to each well with gentle mixing. The absorbance was done within 30 min after the addition of stop solution at 450 nm in Bioteck-XLD800 Multi Scan Plate Reader (Bioteck, VA, USA), and the absorption intensity was inversely proportional to samples SMZ contents. SMZ sheet supplied with the kit was used to generate a standard curve and to calculate the concentration of SMZ in the samples.

### Exposure assessment of SMZ and estimated daily intake (EDI)

The EDI values of SMZ (µg/kg body weight [b.w.]/day) were calculated from the average SMZ level (µg/g) in poultry meat samples, the daily intake of poultry meat, and mean b.w. for adults in Jordan (85 g and 79 kg, respectively) (27 DOS, 2017). The EDI was calculated according to Awaisheh *et al*. [28]:

EDI (µg/kg b.w./day) = [SMZ] × [Daily poultry consumption]/[b.w.].

### Statistical analysis

The statistical analysis has been conducted using SPSS (Version 11.5, SPSS Inc., Chicago, IL, USA). A two-way analysis of variance was used to evaluate the variation in SMZ residue concentrations among different sample sources. p<0.05 was considered to indicate statistical significance.

## Results

### Levels of SMZ residues in different poultry meat samples

In the current study, contamination levels of SMZ in 120 poultry meat samples (60 fresh and 30 frozen domestic and 30 frozen imported samples) were screened. As shown in Table-2, the results revealed that 20 of 120 samples (16.7%) were contaminated with SMZ levels exceeded the international MRL (100 µg/kg), and these samples were considered as violative-positive samples. The average of SMZ was 235.58 µg/kg and ranged from 11.47 to 810 µg/kg. In detail, 11 samples (18.3%) and 1 sample (3.3%) of the fresh and frozen domestic poultry samples were contaminated with SMZ above the MRL, with average of 265.07 and 66.87 µg/kg, and range of 11.47-810 µg/kg for both types of samples, respectively (Table-2). Whereas, 8 samples (26.7%) of the frozen imported poultry samples were contaminated with the highest level of SMZ, with an average of 353.08 µg/kg, and range of 11.47-810 µg/kg. However, 51 samples (42.5%) were found to have SMZ levels between 10 and 100 µg/kg and these samples were considered as non-violative-positive samples; and 49 samples (40.8%) were found to have SMZ levels below the detection limit of the ELISA kit (<10 µg/kg) and these samples were considered as negative samples.

**Table-2:** Numbers (%) of sulfamethazine negative, non-violative positive, and violative positive in poultry samples.

Poultry source	Negative^1^ (%)	Non-violative positive^2^ (%)	Violative positive^3^ (%)	Average (µg/kg)^4^	Minimum-maximum results	Total
Domestic–Fresh	27 (45)	22 (36.7)	11 (18.3)	265.07^b,5^±14.53	11.47->810	60
Domestic–Frozen	11 (36.7)	18 (60)	1 (3.3)	66.87^c^±2.98	11.47->810	30
Imported–Frozen	11 (36.7)	11 (36.7)	8 (26.7)	353.08^a^±18.37	11.47->810	30
Total	49 (40.8)	51 (42.5)	20 (16.7)	235.58±15.83	11.47->810	120

1 Negative samples=Samples with no detected antibiotic,

2 Non-violative-positive sample=Samples with detected antibiotic below MRL (100 ppb), and

3 Violative positive=Samples with antibiotic exceeding the MRL.

4 Results are means±SE of four determinations.

5 Means within the same row with different letters are significantly different (p<0.05). SE=Standard error, MRL=Maximum residue limit

### Exposure assessment of SMZ and EDI

Up to our best knowledge, this is also the first-ever study that assessed the EDI of SMZ by Jordanian adults, and one of the very few in the world assessed the international EDI of SMZ. Table-3 showed that the average EDI of SMZ was 0.286 µg/kg b.w./day. Results revealed that the highest EDI came from imported frozen poultry with average of 0.429 µg/kg b.w., followed by domestic fresh poultry with average of 0.322 µg/kg b.w./day, and the lowest EDI was for domestic frozen poultry with average of 0.081 µg/kg b.w/day.

**Table-3:** EDI of SMZ by Jordanian population.

Poultry source	SMZ residues level (µg/kg)	Daily poultry meat intake (g)	Average b.w. (kg)	EDI (µg/kg b.w./day)^1^
Domestic–Fresh	265.07	85	70	0.322^b2^±0.021
Domestic–Frozen	66.87	85	70	0.081^c^±0.007
Imported–Frozen	353.08	85	70	0.429^a^±0.047
Average	235.58	85	70	0.286±0.032

1 Results are means±SE.

2 Means within the same row with different letters are significantly different (p<0.05). EDI=Estimated daily intake, SMZ=Sulfamethazine, b.w.=Body weight, SE=Standard error

## Discussion

### Incidence rate and contamination levels of SMZ in poultry meat

The current research is the first-ever study reporting the SMZ levels in poultry meat in Jordan. SMZ is a suspected carcinogen and its presence in food could expose a great health risk to human. Furthermore, the incidence of SMZ has been confirmed in various food types including meat, fish, milk, and cheese products [21,27,29]. In addition, SMZ is the major cause of approximately 95% of all SAs violations in animal tissues [17]. However, the current study revealed that 16.7% of the surveyed samples exceeded the MRL with an average of 235.58 µg/kg and considered as unfit for human consumption. The highest incidence rate and contamination level were observed in frozen imported samples (26.7% and 353.05 µg/kg, respectively). The same pattern but with less incidence rate and contamination level was observed in fresh domestic samples (18.3% and 265.07 µg/kg, respectively). Unexpectedly, the frozen domestic samples showed the lowest incidence rate and contamination level (3.3% and 66.87 µg/kg, respectively). This high incidence rate and contamination level of SMZ in both domestic and imported poultry samples may indicate the high using rate of SMZ in the intensive poultry production system, which is likely due to SMZ high efficacy in fighting diseases and promoting growth in poultry.

In Jordan, SMZ incidence in poultry meat had not been reported before, and on extensive search, only one study was found about the incidence of four SAs, including SMZ, in red meat in Jordan [30]. This study reported that 3 of 36 sheep and beef meat samples contained detectable but below the MRL levels of SAs. Internationally, a limited number of studies concerning the incidence of SMZ in poultry meat was found. However, The SMZ levels detected in poultry samples in Jordan were comparable to those reported in many other studies. In agreement to our results, Mehtabuddin *et al*. [23] surveyed the contamination level of SAs including SMZ in poultry meat samples in Pakistan and reported that 23% of the samples exceeded the MRL and were unfit for human consumption, with the range of 0.02-0.8 µg/g. Another study in Lebanon reported that 1 of 80 samples contained 17.3 µg/kg of SMZ in poultry meat [31]. Moreover, Cheong *et al*. [21] reported that, among different SAs, SMZ residues in poultry meat in Malaysia ranged from 7 to 39 µg/kg. In general, it is very common to survey SMZ incidence in different food products including poultry meat as part of the surveying SAs group. For instance, in the USA, the SAs incidence rates were reported to over 4%, while in Italy, a lower incidence rate was reported (< 1% violation) [32]. Another study in Nigeria on the occurrence of veterinary drug residues, including SAs, in poultry products showed contamination of 33.1% in broilers [33]. In agreement with the results reported in the current study, Salem [34] and Shaikh and Chu [35] found SA residues above MRL level in chicken meat samples.

Even though there was no report about SMZ incidence in Jordan or many other Middle East countries for more realistic comparison, it is quite obvious and crucial that the contamination of poultry meat with antibiotic residues including SMZ is an international problem. Therefore, the protection of consumers from SMZ residues requires fundamental national and international collaboration and far-reaching agreements of national and international regulations to control antibiotics usage in animal production systems. Moreover, the current findings advise that the best strategy to control antibiotics residue in animal, particularly in poultry meat, should start at the farm level, through strict control and monitoring system of using antibiotics in animal production and applying the good agricultural practices for proper observation of antibiotics withdrawal period. Moreover, several natural plant and probiotics bacterial extracts with strong antibacterial effects have been investigated recently to control many foodborne pathogens in meat and meat products [36-39]. These natural extracts could represent promising alternatives of antibiotics in poultry production to maintain a low mortality rate, a good level of animal yield while preserving environment and consumer health.

### EDI of SMZ by Jordanian adults

Up to our best knowledge, this is the first research that evaluated the EDI of SMZ by Jordanian adults from poultry meat. Even though there is no tolerable daily intake recommended by regulatory authorities for exposure to SAs, including SMZ, the present data seemed to reveal high EDI values (Table-3). Furthermore, the previous studies on SAs and SMZ exposure from other countries are very scarce, and only one study by Cheong *et al*. [21] that evaluated the EDI of SAs including SMZ was found to compare with. In that study, the exposure of SAs from chicken consumption in Malaysian consumers ranged from 0.002 to 0.088 (µg/kg b.w./day) [21]. Compared to these results, it is worthy to note that the Jordanian EDI of SMZ is very high. The serious and important issues emerge here are the high-risk possibility of Jordanian population exposure to SMZ from poultry in Jordan and the world.

## Conclusion

The current data represented the first study of SMZ level and EDI in poultry meat in Jordan. Of the 120 surveyed samples, 20 (16.7%) were SMZ violative positive and exceeded the EU maximum limit. The maximum SMZ values detected were 353.08 and 265.07 µg SMZ/kg of frozen imported and fresh domestic poultry meat, respectively. It is also noteworthy the high EDI of SMZ by Jordanian adults (0.286 µg SMZ/kg b.w./day). The findings of the current study indicated high SMZ incidence rate, contamination level, and EDI values in imported and domestic poultry meat, accordingly raised the need to reinforce good agricultural practices as a prophylactic and control measures to control SMZ level in poultry meat through strict use of antibiotics in animal production and proper observation of antibiotics withdrawal period by applying the good agricultural practices.

## Authors’ Contributions

SSA, MSK, and RJR: designed and conducted the research, collection, and analyzed the data. RMA: manuscript writing; JMA: manuscript writing and reviewing. RJR: references writing and reviewing. All authors read and approved the final manuscript.

## References

[1] Chattopadhyay M.K (2014). Use of antibiotics as feed additives:A burning question. Front. Microbiol.

[2] Emami K, Samie A, Rahmani H.R, Ruiz-Feria C.A (2012). The effect of peppermint essential oil and fructooligosaccharides, as alternatives to virginiamycin, on growth performance, digestibility, gut morphology and immune response of male broilers. Anim. Feed Sci. Tech.

[3] De Briyne N, Atkinson J, Pokludová L, Borriello S.P (2014). Antibiotics used most commonly to treat animals in Europe. Vet. Rec.

[4] Adebowale O.O, Adeyemo O.K, Awoyomi O, Dada R, Adebowale O (2016). Antibiotic use and practices in commercial poultry laying hens in Ogun state Nigeria. Rev. Elev. Med. Vet. Pays Trop.

[5] Garofalo C, Vignaroli C, Zandri G, Aquilanti L, Bordoni D, Osimani A, Biavasco F (2007). Direct detection of antibiotic resistance genes in specimens of chicken and pork meat. Int. J. Food Microbiol.

[6] Manyi-Loh C, Mamphweli S, Meyer E, Okoh A (2018). Antibiotic use in agriculture and its consequential resistance in environmental sources:Potential public health implications. Molecules.

[7] Dantas G, Sommer M.O, Oluwasegun R.D, Church G.M (2008). Bacteria subsisting on antibiotics. Science.

[8] Martínez J.L (2008). Antibiotics and antibiotic resistance genes in natural environments. Science.

[9] SANCO (2004). The Directorate General for Health and Consumer Affairs Report for 2003 on the Results of Residue Monitoring in Food of Animal Origin in the Member State (SANCO/2810/2004).

[10] Bhushan C, Khurana A, Sinha R, Nagaraju M (2017). Antibiotic Resistance in Poultry Environment:Spread of Resistance from Poultry Farm to Agricultural Field.

[11] Patel T, Marmulak T, Gehring R, Pitesky M, Clapham M.O, Tell L.A (2018). Drug residues in poultry meat:A literature review of commonly used veterinary antibacterials and anthelmintics used in poultry. J. Vet. Pharm. Ther.

[12] World Health Organization (2015). Global Action Plan on Antimicrobial Resistance.

[13] Wielinga P.R, Schlundt J, Food safety:At the center of a one health approach for combating zoonoses (2012). One Health:The Human-Animal-Environment Interfaces in Emerging Infectious Diseases.

[14] Hela W, Brandtner M, Widek R, Schuh R (2003). Determination of sulfonamides in animal tissues using cation exchange reversed phase sorbent for sample cleanup and HPLC-DAD for detection. Food Chem.

[15] Barceló D (2007). Pharmaceutical-residue analysis. Trends Anal. Chem.

[16] Van Boeckel T.P, Brower C, Gilbert M, Grenfella B.T, Levina S.A, Robinsoni T.P, Laxminarayan R (2015). Global trends in antimicrobial use in food animals. Proc. Nat. Acad. Sci. U. S. A.

[17] Chen J, Zhou X, Zhang Y, Gao H (2012). Potential toxicity of sulfanilamide antibiotic:Binding of sulfamethazine to human serum albumin. Sci. Total Environ.

[18] Awaisheh S.S (2010). Incidence and contamination level of *Listeria monocytogenes* and other *Listeria* spp in ready-to-eat meat products in Jordan. J. Food Prot.

[19] Awaisheh S.S (2009). Survey of *Listeria monocytogenes* and other *Listeria* sp contamination in different common ready-to-eat food products in Jordan. Pak. J. Biol. Sci.

[20] Baynes R.E, Dedonder K, Kissell L, Mzyk D, Marmulak T, Smith G, Riviere J.E (2016). Health concerns and management of select veterinary drug residues. Food Chem. Toxicol.

[21] Cheong C.K, Hajeb P, Jinap S, Ismail-Fitry M.R (2010). Sulfonamides determination in chicken meat products from Malaysia. Int. Food Res. J.

[22] Gehring T.A, Griffin B, Williams R, Geiseker C, Rushing L.G, Siitonen P.H (2006). Multiresidue determination of sulfonamides in edible catfish, shrimp and salmon tissues by high-performance liquid chromatography with postcolumn derivatization and fluorescence detection. J. Chromatogr. B Analyt. Tech. Biomed. Life Sci.

[23] Mehtabuddin A, Ahmad T, Nadeem S, Tanveer Z, Arshad J (2012). Sulfonamide residues determination in commercial poultry meat and eggs. J. Anim. Plant Sci.

[24] Mubito E.P, Shahada F, Kimanya M.E, Buza J.J (2014). Sulfonamide residues in commercial layer chicken eggs in Dar-es-Salaam, Tanzania. Am. J. Res. Commun.

[25] Liman B.C, Kanbur M, Eraslan G, Baydan E, Dinį E, Karabacak M (2015). Effects of various freezing and cooking processes on the residues of sulfamethazine in broiler tissues. Vet. Fakült. Derg.

[26] Regulations EU (1990). Council Regulation (EEC) No 2377/90 of 26 June 1990 Laying Down a Community Procedure for the Establishment of Maximum Residue Limits of Veterinary Medicinal Products in Foodstuffs of Animal Origin. Official J. Eur. Communities.

[27] Department of Statistics (2017). Jordan Statistical Yearbook.

[28] Awaisheh S.S, Rahahleh R.J, Algroom R.M, Al-Bakheit A.A, Al-Khaza'leh J.M, Al-Dababseh B.M (2019). Contamination level and exposure assessment of aflatoxin M1 in infant milk formula by Jordanian infants. Ital. J. Food Saf.

[29] Wen Y, Zhang M, Zhao Q, Feng Y.Q (2005). Monitoring of five sulfonamide antibacterial residues in milk by in-tube solid-phase microextraction coupled to high-performance liquid chromatography. J. Agric. Food Chem.

[30] Alawi M, Othman M.A, Ahmad R (2014). Levels of sulfonamides in local food of animal origin (muscle tissues, kidney and liver) using HPLC-PDA. Eur. Int. J. Appl. Sci. Technol.

[31] Jammoul A, El Darra N (2019). Evaluation of antibiotics residues in chicken meat samples in Lebanon. Antibiotics.

[32] Dey B.P, Thaler A, Gwozdz F (2003). Analysis of microbiological screen test data for antimicrobial residues in food animals. J. Environ. Sci. Health Part B.

[33] Kabir J, Umoh V.J, Audu-Okoh E, Umoh J.U, Kwaga J.K.P (2004). Veterinary drug use in poultry farms and determination of antimicrobial drug residues in commercial eggs and slaughtered chicken in Kaduna State, Nigeria. Food Control.

[34] Salem D.A (2004). Monitoring of some antimicrobial residues in chicken from Assiut, Egypt.

[35] Shaikh B, Chu P.S (2000). Distribution of total 14C residue in egg yolk, albumen, and tissues following oral [14C] sulfamethazine administration to hens. J. Agric. Food Chem.

[36] Awaisheh S.S (2013). Efficacy of fir and qysoom essential oils, alone and in combination, in controlling *Listeria monocytogenes in vitro* and in RTE meat products model. Food Control.

[37] Awaisheh S.S, Ibrahim S.A (2009). Screening of antibacterial activity of lactic acid bacteria against different pathogens found in vacuum packaged meat products. Foodborne Pathog. Dis.

[38] Mehdi Y, Létourneau-Montminy M.P, Gaucher M.L, Chorfi Y, Suresh G, Rouissi T, Brar S.K, Côté C, Ramirez A.A, Godbout S (2018). Use of antibiotics in broiler production:Global impacts and alternatives. Anim. Nutr.

[39] Awaisheh S.S, Al-Nabulsi A.A, Osaili T.M, Ibrahim S, Holley R (2013). Inhibition of *Cronobacter sakazakii* by heat labile bacteriocins produced by probiotic LAB isolated from healthy infants. J. Food Sci.

